# Hemoadsorption during Cardiopulmonary Bypass in Patients with Endocarditis Undergoing Valve Surgery: A Retrospective Single-Center Study

**DOI:** 10.3390/jcm10040564

**Published:** 2021-02-03

**Authors:** David Santer, Jules Miazza, Luca Koechlin, Brigitta Gahl, Bejtush Rrahmani, Alexa Hollinger, Friedrich S. Eckstein, Martin Siegemund, Oliver T. Reuthebuch

**Affiliations:** 1 Department of Cardiac Surgery, University Hospital Basel, 4031 Basel, Switzerland; david.santer@usb.ch (D.S.); jules.miazza@usb.ch (J.M.); luca.koechlin@usb.ch (L.K.); brigitta.gahl@usb.ch (B.G.); bejtush.rrahmani@usb.ch (B.R.); friedrich.eckstein@usb.ch (F.S.E.); 2 Department of Intensive Care Medicine, University Hospital Basel, 4031 Basel, Switzerland; alexa.hollinger@usb.ch (A.H.); martin.siegemund@usb.ch (M.S.); 3 Department of Clinical Research, University Hospital Basel, 4031 Basel, Switzerland

**Keywords:** endocarditis, cardiopulmonary bypass, hemoadsorption, Cytosorb, blood purification, sepsis, cardiac surgery, valve surgery

## Abstract

Background: Aim of this study was to evaluate the outcomes of endocarditis patients undergoing valve surgery with the Cytosorb^®^ hemoadsorption (HA) device during cardiopulmonary bypass. Methods: From 2009 until 2019, 241 patients had undergone valve surgery due to endocarditis at the Department of Cardiac Surgery, University Hospital of Basel. We compared patients who received HA during surgery (*n* = 41) versus patients without HA (*n* = 200), after applying inverse probability of treatment weighting. Results: In-hospital mortality, major adverse cardiac and cerebrovascular events and postoperative renal failure were similar in both groups. Demand for norepinephrine (88.4 vs. 52.8%; *p* = 0.001), milrinone (42.2 vs. 17.2%; *p* = 0.046), red blood cell concentrates (65.2 vs. 30.6%; *p* = 0.003), and platelets (HA vs. Control: 36.7 vs. 9.8%; *p* = 0.013) were higher in the HA group. In addition, a higher incidence of reoperation for bleeding (34.0 vs. 7.7 %; *p* = 0.011), and a prolonged length of in-hospital stay (15.2 (11.8 to 19.6) vs. 9.0 (7.1 to 11.3) days; *p* = 0.017) were observed in the HA group. Conclusions: No benefits of HA-therapy were observed in patients with infective endocarditis undergoing valve surgery.

## 1. Introduction

Patients undergoing valve surgery due to infective endocarditis (IE) are heterogenous, yet they present with a persistently high perioperative mortality, ranging from 7.6 to 25% [1,2,3]. Even if the patients receive optimal antibiotic treatment nowadays, postoperative sepsis is still the main reason for adverse outcomes [4]. The biocompatibility of cardiopulmonary bypass (CPB) has undergone constant improvements in recent years [5], since efficient regimens to treat septic shock, especially in endocarditis patients, are of paramount importance. Patients suffering from IE are at higher risk for complications such as stroke, heart failure, or in-hospital mortality compared to patients undergoing cardiac surgery without IE [6], since they are in a higher inflammatory state [7,8]. It was shown that patients undergoing cardiac surgery with CPB that present with higher levels of preoperative inflammatory markers are also more prone to postoperative complications such as low cardiac output syndrome and cardiac death [9].

Possibly, patients producing higher inflammatory mediator levels have suffered more severe valvular damages by IE than patients revealing lower inflammatory mediator levels. In addition, high serum levels of inflammatory mediators might reflect an insufficient control of the infection, which could lead to complications (e.g., multi-organ failure, myocardial failure, etc.) [7,9]. Interleukin-6 peak levels were shown to correlate with aortic cross clamp time as well as postoperative myocardial dysfunction [10]. As a consequence, the potential of blood purification during CPB to reduce inflammatory mediators was investigated, yet with controversial results [11,12,13].

The CytoSorb^®^ (HA, Cytosorbents Corporation, NJ, USA) hemoadsorption device is an extracorporeal cytokine adsorber that was designed to remove inflammatory mediators in critically ill patients. It consists of polymer beads that bind compounds in the range of 10 to 55 kDa and is installed into the venous system of the CPB between the oxygenator and the reservoir. Besides decreasing the inflammatory response, HA might even reduce bleeding complications in patients who undergo emergency cardiac surgery with ticagrelor or rivaroxaban [10]. Promising case series in critically ill patients reported that HA is safe and not associated with adverse events [7,8,14]. One unmatched retrospective study in patients with mitral valve IE reported a reduced demand of vasopressors and a lower incidence of postoperative sepsis [15]. However, no study has shown significant clinical benefits in patients undergoing cardiac surgery to date. To the best of our knowledge, this is the first work based on a retrospective inverse probability of treatment-weighted analysis comparing the effects of HA in IE patients undergoing valve surgery with CPB. Aim of this study was the evaluation of clinical benefits of HA therapy with in-hospital mortality as primary outcome measure. The effect of HA on cytokine levels was not analyzed and, due to the nature of this retrospective study, not part of the study design.

## 2. Materials and Methods

**Figure 1:**
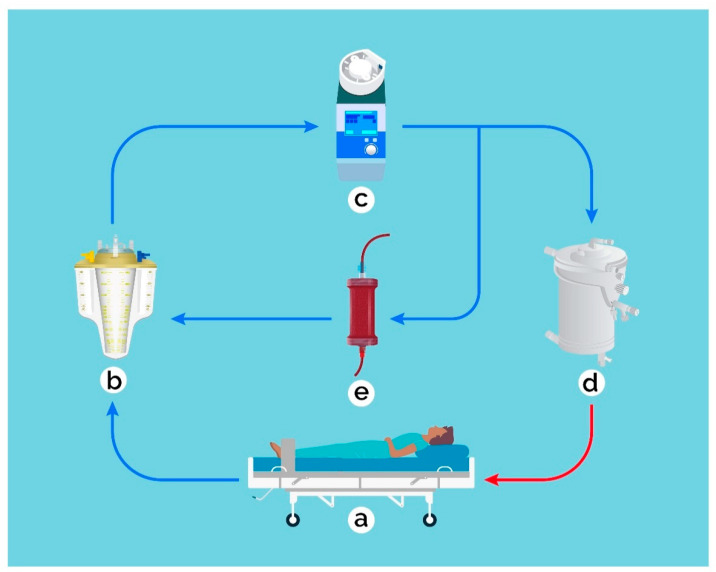
The hemoadsorption (HA) device was installed into the venous cardiopulmonary bypass, so that the blood (blue: venous, red: arterial) was pumped via a side arm back into the reservoir. The average flow rate via the HA device was 500 mL/min. (**a**) patient, (**b**) venous reservoir, (**c**) roller pump, (**d**) oxygenator, (**e**) HA device.

We performed a retrospective single-center database analysis at the Department of Cardiac Surgery, University Hospital of Basel, Switzerland. A total of 241 patients (>18 years) had undergone cardiac surgery for IE between January 2009 and December 2019. Hemoadsorption during CPB was introduced in Basel in 2016 and has ever since been used in most endocarditis patients during valve surgery. The HA device was installed into the venous CPB, so that the blood was pumped via a side arm back into the reservoir, as described previously (Figure 1) [15]. The average flow rate via the HA device was 500 mL/min. Hemoadsorption was discontinued at the ICU, for example during continuous veno-venous hemofiltration treatment. Endocarditis was diagnosed according to the Duke criteria [16] in all patients. Two groups were formed: patients who were treated with HA during CPB were retrospectively assigned to the HA-group, all other patients served as control group. Patient characteristics, inclusion criteria, risk factors, surgical details, and outcome data are routinely collected in the department’s prospectively maintained quality management software (Dendrite Clinical Systems, V1.7), and regularly checked for completeness and consistency. Data from the intensive care unit (ICU) were analyzed for the first 24 postoperative hours. Inotropy and blood product demand during reoperations within the first 24 h were included into the analyses. Major adverse cardiac and cerebrovascular events (MACCE) were defined as in-hospital mortality, myocardial ischemia or stroke and serological parameters. Neurological complications included ischemic events, encephalopathy, meningitis, hemorrhages, and brain abscesses. According to standard protocol, no heparin was administered during the first six hours after arrival at the ICU. The study was conducted in accordance with the Declaration of Helsinki, and the protocol was approved by the local Ethics Committee of Northwestern and Central Switzerland (BASEC Req-2019-01740). The trial was registered at ClinicalTrials.gov (www.clinicaltrials.gov (accessed on 20 December 2020), identifier: NCT04309591).

### Statistical Analysis

To investigate the impact of HA on the outcome, we used inverse probability of treatment weighting (IPTW) in order to achieve balanced distributions of baseline characteristics in both treatment groups, and to minimize confounding by indication. We included the perioperative intake of platelet aggregation inhibitors, European System for Cardiac Operative Risk Evaluation (EuroSCORE II) score after log-transformation, patient age, New York Heart Association Functional Classification (NYHA) class III or IV, prior myocardial infarction, peripheral artery disease, and nicotine use as covariates into the propensity model. We truncated IPT weights that exceeded the 1st or 99th percentile [17]. As balance diagnostics, we calculated standardized differences of pre-treatment variables. Absolute values of standardized differences of 0.1 or less were considered to indicate no relevant difference between treatment groups. (Scatterplot in Figure S1) We used mixed linear models after IPTW to study whether HA impacts the development of hemoglobin, fibrinogen, C-reactive protein (CRP), platelets, and white blood cell (WBC) counts within five days after surgery, including an interaction term HA × time. In order not to depend on linearity of marker development, we repeated the analysis only including measurements of day one and two, and day one, two, and three, respectively. During the period of patient enrollment, intensive care strategies underwent some relevant changes in our hospital. Fluid resuscitation at the ICU was changed in 2016 to less fluid and increased inotropic support and fresh frozen plasma was used to replace for hydroxyethyl starch since 2014. To account for these changes, we adjusted for impact of time using fractional polynomials as a sensitivity analysis, and report adjusted *p*-values for variables which might be substantially affected by these changes: epinephrine, dobutamine, milrinone, norepinephrine, and fresh frozen plasma (FFP). Continuous variables were presented as mean ± standard deviation if normally distributed, or as geometric mean with standard deviations back-transformed from the log scale if distribution was skewed. Corresponding *p*-values were calculated using linear regression on the variable or on the log-transformed variable. We dichotomized medical intensive care treatment details due to skewed distribution, zero inflation, and the small sample size. Categories were presented as numbers and percentage, *p*-values were calculated using logistic regression for binary variables, or multinomial regression otherwise. Significance was accepted at *p* < 0.05. Statistical analyses were performed with Stata 15 (StataCorp, College Station, TX, USA).

## 3. Results

### 3.1. Patient Characteristics

A total of 241 patients had undergone cardiac surgery for IE at the University Hospital Basel between 2009 and 2019. Patient characteristics are displayed in Table 1. Forty-one of these patients (17%) had received perioperative HA. Patient characteristics were similar in both groups: preoperative incidence of intake of a platelet aggregation inhibitor (*p* = 0.389), stroke (*p* = 0.302; Table 1), rate of emergency procedures (*p* = 0.850; Table 1), and EuroSCORE II (*p* = 0.185; Table 1) were comparable among the two groups. The most common microbiological etiology of IE was *Staphylococcus aureus* in the HA group (34.2%), and *Streptococcus viridans* (22.3%) in the control group (Supplementary Table S1). Preoperative blood work was comparable in both groups (Table 1). Patient characteristics before IPTW are shown in Supplementary Table S2.

### 3.2. Perioperative Data

Perfusion (HA vs. Control: 110 (80 to 150) vs. 138 (130 to 146) min; *p* = 0.327) as well as aortic clamping time (HA vs. Control: 92.7 ± 83.1 vs. 106.4 ± 48.3 min; *p* = 0.308) were similar in both groups. There was neither a difference in the use of intravenous inotropes prior surgery (*p* = 0.487), nor in type of surgery (Table 2). Perioperative data before IPTW are shown Supplementary Table S3.

### 3.3. Intensive Care Unit Data

Markedly, more patients in the HA group required norepinephrine (HA vs. Control: 88.4 vs. 52.8 %; *p* = 0.001) and milrinone (HA vs. Control 42.2 vs. 17.2%; *p* = 0.046; Table 3). Demand for epinephrine (*p* = 0.365), dobutamine (*p* = 0.612), and nitroglycerine (*p* = 0.104) was comparable between the two groups (Table 3). Length of stay at the ICU was prolonged in the HA group (5.1 (3.8 to 6.8) vs. 3.2 (2.7 to 3.8) days; *p* = 0.230), yet without significance. Besides the markedly increased demand for red blood cell concentrates (HA vs. Control: 60.3 vs. 30.5%; *p* = 0.003) and platelets (HA vs. Control: 36.7 vs. 9.8%; *p* = 0.013), we observed a tendency towards more patients receiving FFP (HA vs. Control: 58.3 vs. 24.6%; *p* = 0.075) in the HA group (Table 3). Tranexamic acid (*p* = 0.497), Haemate (Haemate P, CSL Behring AG, Bern, Switzerland, *p* = 0.241), fibrinogen (*p* = 0.194), and prothrombin complex (Prothromplex NF 600 IE, Takeda Pharma AG, Glattpark, Switzerland, *p* = 0.489) were administered in similar amounts in both groups (Table 3). Drainage volume > 800mL within 12 h was observed more often in the HA group, yet without significance (HA vs. Control: 43.7 vs. 23.8%; *p* = 0.128; Table 3). The rate of reoperation for bleeding was significantly increased in the HA group (HA vs. Control: 34.0 vs. 4.8 %; *p* = 0.011; Table 3). Incidence of prolonged intubation >72 h was similar in both groups (*p* = 0.138; Table 3). Intensive care unit data before IPTW are shown Supplementary Table S4.

### 3.4. Postoperative Results

In-hospital mortality (*p* = 0.485), incidence of delirium (*p* = 0.095), MACCE (*p* = 0.704), neurological complications (*p* = 0.110), postoperative renal failure (*p* = 0.360), postoperative pulmonary infection (*p* = 0.782), and atrial fibrillation at discharge (*p* = 0.129) were comparable between the two groups (Table 4). The duration of hospital stay was significantly longer in the HA group than in the control group (HA vs. control: 15.2 (11.8 to 19.6) vs. 9.0 (7.1 to 11.3) days; *p* = 0.017, Table 4). The incidence for postoperative permanent pacemaker implantation was higher in the control group (HA vs. control: 5.6 vs. 19.6 %; *p* = 0.021; Table 4). Postoperative data before IPTW are shown Supplementary Table S5.

### 3.5. Laboratory Analysis

**Figure 2:**
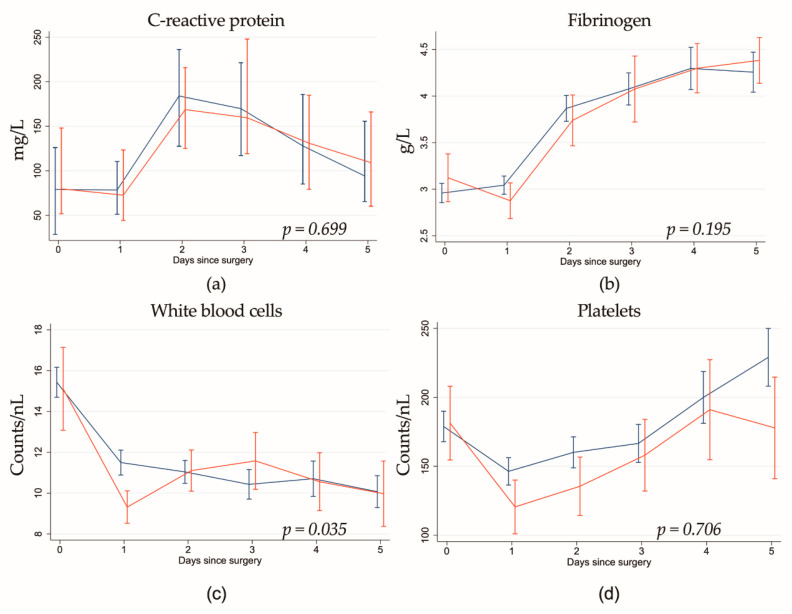
Postoperative development of (**a**) C-reactive protein, (**b**) fibrinogen, (**c**) white blood cells, and (**d**) platelets in hemoadsorption (red) vs. control (blue) group. White blood cell counts were decreased on the first postoperative day in the hemoadsorption group, which evened out until day five. Hemoadsorption did not show any further association with any other marker, and we could not observe any interaction of hemoadsorption and time. Given *p*-values are for interaction.

While preoperative levels were comparable between the two groups, we found a significant decrease of WBC counts on the first five postoperative days in patients who had undergone HA treatment (Table 5, Supplementary Table S6, Figure 2). Hemoadsorption did not show any association with other markers (hemoglobin, C-reactive protein, fibrinogen, platelets).

## 4. Discussion

This retrospective single-center study describes the outcome after HA application during CPB in IE patients undergoing valve surgery. While perioperative mortality and length of ICU stay were statistically comparable in both groups, the in-hospital stay was significantly longer in HA group. The most important finding of this study is that HA patients were associated with higher reoperation rates for bleeding than the patients in the control group. In addition, demand for norepinephrine, red blood cell concentrates, and platelets was markedly increased in the HA group.

In-hospital mortality in patients with IE undergoing valve surgery ranges from 7.6 to 25% [1,2,3]. Träger et al. [14] reported in-hospital mortality rates of 25% in 39 IE patients treated with HA perioperatively, which was higher than in our study. An explanation might be that, compared to our studied patients, the patient population in this study was more heterogenous (mean EuroSCORE II: 11 (2.2–96.7)%; mean CPB time: 132 (64–445) min). Two small single-center randomized controlled trials [18,19] comparing the cytokine profiles and clinical outcomes of patients undergoing elective cardiac surgery showed no influence of HA on short-term mortality [18]. This is in line with our data, which show comparable in-hospital mortality rates for both the HA and the control group.

Hemoadsorption with Cytosorb^®^ targets molecules with a molecular weight from 10–60 kDa [20], a range which also includes coagulation factors, such as protein C, antithrombin III (58 kDa), Factors VII (50 kDa) and X (58.8 kDa). While previous studies have reported a relevant platelet drop in patients treated with HA [21,22], a recent retrospective study by Hassan et al. [23] on emergency open-heart surgery suggested that HA might reduce postoperative bleeding, drainage, and rethoracotomy rates in patients undergoing surgery with either Ticagrelor or Rivaroxaban. These results might be partly explained by a preclinical study that showed a >99% elimination of Ticagrelor in human blood experiments [24]. However, besides its small sample size, the trial by Hassan et al. also lacks a matched control [23]. In our control group, the reoperation rate for bleeding was consistent with the one reported in the existing literature [25,26]. However, an almost four-fold increased rate of reoperations for bleeding was observed in the HA group. Furthermore, the postoperative demand for blood products (red blood cell concentrates and platelets) was significantly higher in patients that had been treated with HA. Consecutively, the increased bleeding caused by adsorption of coagulation factors probably also led to hypovolemia in the HA group, which is another thinkable explanation for our observation of higher milrinone and norepinephrine support. Interestingly, although the fluid resuscitation protocol has been changed in 2016 from high volume and low inotropic support to low volume and high inotropic support at our institution, the HA group still showed an increased demand for norepinephrine and milrinone, even after adjustment for time. HA during CPB has been introduced at our department in 2016. Since postoperative hemodilution has been higher before 2014, we would have expected an increased rate of red blood cell concentrates (RBC), platelets, and reoperation for bleeding in the control group, if both groups were similar. To the best of our knowledge, no other study has shown an increased reoperation rate due to bleeding in the context of HA to date.

Next to the potential adsorption of coagulation factors, the higher demand for inotropic support might be explained by induction of vasoplegia due to HA, which affects the levels of various endogenous vasoconstrictors: HA reduces (1) cortisol in brain-dead subjects [27,28], (2) thromboxane levels in an ex-vivo model in porcine kidneys [29], (3) Big-Endothelin-1, a precursor of vasoconstricting endothelin-1, that peaks at the onset of sepsis and is up-regulated by interleukin-1, interleukin-2 and interleukin-6 [30], and (4) albumin, which maintains the plasma colloid oncotic pressure [31] and might improve hemodynamics after cardiac surgery [21,32] (Table 6). Further studies are needed to evaluate the eventual vasoplegic impact of HA via adsorption of these regulators.

Recent unmatched reports suggested a reduction of the duration of the ICU stay in HA-treated patients [9,10], as well as potential financial savings by the use of HA [37], which could not be confirmed in our patient cohort. In our study, the HA group presented a prolonged length of in-hospital stay and a higher demand for blood products.

Data on the use of HA in cardiac surgery are scarce, and the existing literature is controversial. A randomized controlled trial (RCT) of 37 patients undergoing cardiac surgery with or without the concomitant use of HA failed to demonstrate a significant reduction of peri- and postoperative levels of pro-inflammatory cytokines and showed no differences in clinical outcomes between the two groups [19]. Another study in patients with endocarditis showed postoperative IL-6 and IL-8 reduction and comparable hemodynamic stability in 39 patients treated with HA [38]. In patients undergoing heart transplantation as well as in patients with severe postoperative SIRS, HA might improve the clinical outcome although all the conducted investigations described only small sample sizes [38,39]. Recently, Haidari et al. [15] described similar postoperative WBC counts, but a reduction of postoperative sepsis in patients with mitral valve IE and HA therapy during CPB (HA vs. Control: 17 vs. 39%, *p* = 0.005). Our results are not consistent with those of Haidari’s study, since although we even observed a significant reduction of WBCs in the HA group, postoperative sepsis has occurred more frequently in the HA group (HA vs. Control 14.4 vs. 6.2 %; *p* = 0.108). According to Bernardi et al. [19], IL-6 expression peaks around 24 h after the patient is taken off the CPB. Therefore, termination of HA administration together with CPB might be too soon to observe any clinical effect.

### Study Limitations

The authors acknowledge three restricted limitations of the study. First, it was a single-center, retrospective study with a limited number of patients. Second, even though IPTW was performed to obtain comparable groups, standardized difference with respect to the intake of platelet inhibitors did not drop below 0.2, indicating residual confounding. However, to the best of our knowledge, this is the first study reporting HA outcomes in valve surgery in patients suffering from IE. Third, inflammatory blood values (e.g., IL-6) are not available for our patients since they are not part of routine laboratory analyses at our department.

## 5. Conclusions

Cardiac surgery performed in IE patients often occurs in an urgent clinical setting. Under such conditions, one is tempted to use every resource available to improve patient outcomes. According to its manufacturer, the administration of HA might be beneficial by removal of inflammatory mediators from the patients’ blood during CPB. In this retrospective study, however, the use of HA did not improve short-term outcomes of IE patients after valve surgery. With this small retrospective study, we cannot provide evidence of reduction in treatment costs, since the rate of reoperation for bleeding and rate of administration of blood products were significantly increased in HA in our study [37]. Further prospective and randomized investigations need to verify whether HA has an effect on coagulation, which might have an impact on reoperation rates and neurological complications. These results, and probably the complexity of IE and sepsis, indicate that future studies need to focus on patient selection as well as ideal timing and extend for HA therapy in IE patients. Additional data from prospective randomized studies [40] are urgently needed to further evaluate the benefits, but also the risks, of this medical device in IE patients. Although the concept of cytokine elimination in endocarditis patients undergoing valve surgery is tempting, a general recommendation for a routine clinical application of HA is still questionable, based on existing literature as well as the results of this study.

## Figures and Tables

**Table 1:** Patient characteristics after inverse probability of treatment weighting (IPTW).

Patient Characteristics	HA (*n* = 41)	Control (*n* = 200)	Stddiff	*p*
Age, years	66.1 ± 23.7	65.4 ± 14.9	0.036	0.854
Female	3 (8.0%)	44 (21.9%)	0.397	0.039
BMI	27.6 ± 11.5	25.9 ± 5.4	0.193	0.345
Ejection fraction, %	55.6 ± 13.3	56.3 ± 10.2	−0.059	0.752
Diabetes	14 (34.5%)	41 (20.6%)	−0.315	0.302
Current Smoker	8 (19.2%)	47 (23.3%)	0.101	0.625
Platelet aggregation inhibitor	19 (46.5%)	115 (57.6%)	0.222	0.389
Peripheral artery disease	3 (6.4%)	16 (8.0%)	0.062	0.705
Preoperative stroke	9 (21.8%)	62 (30.9%)	0.206	0.302
Renal disease	13 (32.5%)	29 (14.4%)	−0.437	0.146
Dialysis	2 (5.7%)	8 (4.2%)	−0.070	0.660
COPD	9 (23%)	16 (7.8%)	−0.430	0.194
Hypertension	20 (48.5%)	104 (52.1%)	0.072	0.793
Hypercholesteremia	17 (40.4%)	64 (31.9%)	−0.179	0.543
NYHA III or IV	20 (48.8%)	87 (43.6%)	−0.104	0.702
Preoperative AF	2 (4.3%)	25 (12.6%)	0.300	0.196
Prior MI	3 (6.1%)	12 (5.8%)	−0.013	0.937
Emergency	5 (12.9%)	23 (11.7%)	−0.036	0.850
EuroSCORE II, %	7.8 (4.8 to 12.5)	8.6 (7.2 to 10.3)	0.219	0.185
CRP, mg/L	1 (0.1 to 14.4)	0.2 (0.1 to 0.4)	0.003	0.076
Fibrinogen, g/L	3.1 (2.8 to 3.4)	2.6 (2.2 to 3.0)	0.508	0.184
Hemoglobin, g/L	72.4 (54.1 to 96.9)	59.3 (43.0 to 81.6)	0.211	0.637
WBC, counts/nL	11.7 (8.3 to 16.5)	9.8 (7.4 to 12.8)	0.242	0.816
Platelets, counts/nL	142 (98 to 207)	107 (76 to 151)	0.190	0.949

HA: hemoadsorption group; stddiff: standardized difference; BMI: body mass index; AF: Atrial fibrillation; COPD chronic obstructive pulmonary disease; NYHA New York Heart Association Functional Classification; MI myocardial infarction; EuroSCORE II European System for Cardiac Operative Risk Evaluation; CRP: C-reactive protein; WBC: white blood cell count.

**Table 2:** Perioperative details after inverse probability of treatment weighting (IPTW).

Perioperative Details	HA (*n* = 41)	Control (*n* = 200)	Stddiff	*p*
Perfusion time, min	110 (80 to 150)	138 (130 to 146)	0.365	0.327
Aortic clamping time, min	92.7 ± 83.1	106.4 ± 48.3	−0.202	0.308
IV inotropes before surgery	6 (14.2%)	39 (19.6%)	0.145	0.487
Aortic valve	33 (81.1%)	143 (71.3%)	−0.230	0.258
Mitral valve	14 (35.3%)	95 (47.5%)	0.251	0.306
Tricuspid valve	1 (2.8%)	11 (5.6%)	0.137	0.393
Severe insufficiency	9 (22.8%)	61 (30.3%)	0.170	0.480
Procedure Groups				0.469
● CABG & Valve(s)	2 (3.7%)	19 (9.6%)	−0.238	
● CABG &Valve(s) & Other	1 (3.5%)	14 (7.2%)	−0.168	
● Valve(s) & Other	16 (38.3%)	75 (37.5%)	0.015	
● Valve(s) only	22 (54.6%)	91 (45.7%)	0.179	
Assist Device				0.141
● IABP	1 (3.0%)	10 (4.9%)	−0.098	
● ECMO	2 (4.1%)	1 (0.4%)	0.247	

HA: hemoadsorption group; stddiff: standardized difference; IV: Intravenous; CABG: Coronary artery bypass grafting; IABP: intraaortic balloon pump; ECMO: extracorporeal membrane oxygenation. Only one patient in the control group underwent concomitant surgery on pulmonary valve; so we did not analyze this variable using IPTW.

**Table 3:** Intensive care unit (ICU) data after inverse probability of treatment weighting.

Intensive Care Unit Data	HA (*n* = 41)	Control (*n* = 200)	Stddiff	*p*
Administration of				
● Epinephrine	23 (57.1%)	89 (44.7%)	−0.250	0.365 *
● Dobutamine	1 (3.5%)	7 (3.6%)	0.006	0.612 *
● Milrinone	17 (42.2%)	34 (17.2%)	−0.567	0.046 *
● Nitroglycerine	2 (4.2%)	29 (14.4%)	0.357	0.057
● Norepinephrine	36 (88.4%)	106 (52.8%)	−0.848	0.001 *
● RBC	27 (65.2%)	61 (30.6%)	−0.738	0.003
● Tranexamic acid	1 (1.9%)	8 (3.9%)	0.122	0.497
● Haemate	3 (7.6%)	7 (3.3%)	−0.190	0.241
● FFP	24 (58.3%)	49 (24.6%)	−0.730	0.075 *
● Fibrinogen	13 (31.2%)	29 (14.6%)	−0.405	0.194
● PCC	4 (8.6%)	25 (12.7%)	0.132	0.489
● Platelets	15 (36.7%)	20 (9.8%)	−0.673	0.013
Intubation >72 h	9 (20.8%)	20 (9.9%)	−0.305	0.406
Drainage >800 mL within 12 h	18 (43.7%)	48 (23.8%)	−0.430	0.128
Length of ICU stay, days	5.1 (3.8 to 6.8)	3.2 (2.7 to 3.8)	0.546	0.230
RRT	3 (6.4%)	13 (6.5%)	0.007	0.970
Reoperation for bleeding	14 (34.0%)	15 (7.7%)	−0.685	0.011
Reoperation later than 24 h	10 (23.6%)	10 (4.8%)	−0.559	0.062

HA: hemoadsorption group; stddiff: standardized difference; RBC: red blood cell concentrates; FFP: fresh frozen plasma; PCC: prothrombin complex concentrate; RRT: renal replacement therapy, * adjusted for impact of time.

**Table 4:** Postoperative outcome after inverse probability of treatment weighting.

Postoperative Details	HA (*n* = 41)	Control (*n* = 200)	Stddiff	*p*
AF at discharge (NOAF)	17 (42.2%)	50 (24.8%)	−0.374	0.129
Delirium	20 (47.9%)	53 (26.7%)	−0.448	0.095
In-hospital mortality	3 (6.8%)	20 (10.0%)	0.118	0.485
Length of hospital stay	15.2 (11.8 to 19.6)	9.0 (7.1 to 11.3)	0.463	0.017
MACCE	5 (11.1%)	27 (13.4%)	0.069	0.704
Neurological complication	20 (49.7%)	58 (29.0%)	−0.433	0.110
Permanent pacemaker	2 (5.6%)	39 (19.6%)	0.432	0.021
Pulmonary infection	4 (9.7%)	17 (8.3%)	−0.049	0.782
Postoperative renal failure	5 (13.2%)	40 (20.0%)	0.185	0.360
Postoperative sepsis	6 (14.4%)	12 (6.2%)	−0.273	0.108
Postoperative stroke	2 (4.3%)	9 (4.3%)	−0.002	0.991
Renal replacement therapy	3 (6.4%)	13 (6.5%)	0.007	0.970

HA: hemoadsorption group; stddiff: standardized difference; AF: atrial fibrillation; MACCE: major adverse cerebrovascular and cardiac events; NOAF: new-onset atrial fibrillation. Neurological complications included ischemic events; encephalopathy; meningitis; hemorrhages; and brain abscesses. Given *p*-values were adjusted for impact of time.

**Table 5:** Association of Cytosorb and biomarker development after inverse probability of treatment weights (IPTW).

Day 1 to 3	HA		Time, Days		Interaction	
Parameter	Coefficient (95% CI)	*p*	Coefficient (95% CI)	*p*	Coefficient (95% CI)	*p*
CRP (mg/L)	−4.82 (−47.7 to 38.1)	0.826	41.8 (35.8 to 47.9)	<0.001	2.88 (−18.5 to 24.2)	0.791
Fibrinogen (g/L)	−0.03 (−0.43 to 0.37)	0.890	0.49 (0.41 to 0.56)	<0.001	0.08 (−0.09 to 0.24)	0.356
Hemoglobin (g/L)	−4.33 (−9.62 to 0.95)	0.108	−2.79 (−3.68 to −1.91)	<0.001	1.36 (−0.87 to 3.59)	0.231
WBC (counts/nL)	−3.94 (−6.33 to −1.55)	0.001	−0.63 (−1.05 to −0.21)	0.003	1.78 (0.62 to 2.93)	0.003
Platelets (counts/nL)	−10.0 (−44.8 to 24.7)	0.572	3.50 (−0.77 to 7.77)	0.108	4.28 (−8.17 to 16.7)	0.500

HA: hemoadsorption group; CI: confidence interval; CRP: C-reactive protein; WBC: white blood cell count.

**Table 6:** Regulators of vasoconstriction reduced by hemoadsorption (HA) and potentially linked to postoperative vasoplegia.

Regulator	Function	Influence of HA
Cortisol	increases response to catecholamines via steroid receptors [33]	↓ [27]
Thromboxane	vasoconstrictor and promoter of platelet aggregation [34]	↓ [35]
Big-endothelin-1	precursor of vasoconstricting Endothelin-1 [30]	↓ [36]
Albumin	Maintenance of plasma colloid oncotic pressure [31] and effect on cardiac preload [32]	↓ [21]

## Data Availability

The datasets used and/or analyzed during the current study are available from the corresponding author on reasonable request.

## Supplementary Materials

The following are available online at https://www.mdpi.com/2077-0383/10/4/564/s1, Figure S1: (a) Scatter plot of standardized differences before and after inverse probability of treatment weighting (IPTW): Patient age, New York Heart Association Functional Classification (NYHA) class III or IV, perioperative intake of platelet aggregation inhibitors, European System for Cardiac Operative Risk Evaluation (EuroSCORE II) score, prior myocardial infarction (MI), peripheral artery disease, and nicotine use (current smoker) as covariates were included into the propensity model. As balance diagnostics, we calculated standardized differences of pre-treatment variables. Absolute values of standardized differences of 0.2 or less were considered to indicate no relevant difference between treatment groups. (b) Kernel Density Plot of the propensity score. Note that probability density has no natural unit, Table S1: Microbiologic etiology: In both groups, the most common microbiologic etiology factors for endocarditis were *Staphylococcus aureus* and the viridans group. Two patients of the control group were infected with two different species, Table S2: Patient characteristics before inverse probability of treatment weighting., Table S3: Perioperative details before inverse probability of treatment weighting. Table S4: Intensive care unit (ICU) data before inverse probability of treatment weighting., Table S5: Postoperative details before inverse probability of treatment weighting., Table S6: Association of hemoadsorption (HA) therapy and biomarker development before inverse probability of treatment weighting. We found that HA only showed an association with a decrease of white blood cell counts on the first postoperative day in patients who had undergone HA treatment, which evened out until day five.
