# Supplementary material for: Hemoadsorption during Cardiopulmonary Bypass in Patients with Endocarditis Undergoing Valve Surgery: A Retrospective Single-Center Study
Source: J Clin Med. 2021 Feb 3;10(4):564. doi: 10.3390/jcm10040564 (PMC7913197; doi:10.3390/jcm10040564)
Supplement: Supplementary file 1 [file jcm-10-00564-s001.pdf]

Journal of
Clinical Medicine

MDPI

Supplementary Material

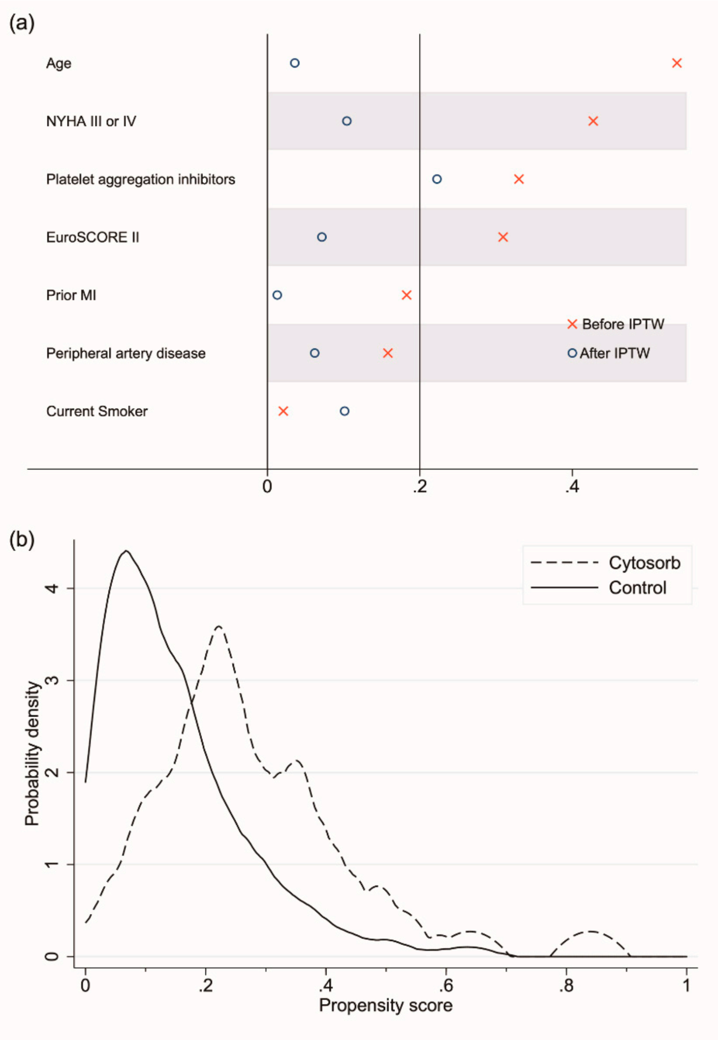

Figure S1. (a) Scatter plot of standardized differences before and after inverse probability of treatment weighting (IPTW): Patient age, New York Heart Association Functional Classification (NYHA) class III or IV, perioperative intake of platelet aggregation inhibitors, European System for Cardiac Operative Risk Evaluation (EuroSCORE II) score, prior myocardial infarction (MI), peripheral artery disease, and nicotine use (current smoker) as covariates were included into the propensity model. As balance diagnostics, we calculated standardized differences of pre-treatment variables. Absolute values of standardized differences of 0.2 or less were considered to indicate no relevant difference between treatment groups. (b) Kernel Density Plot of the propensity score. Note that probability density has no natural unit.

Table S1. Microbiologic etiology: In both groups, the most common microbiologic etiology factors for endocarditis were staphylococcus aureus and the viridans group. Two patients of the control group were infected with two different species.

Hemoadsorption	*n* (%)	Control	*n* (%)	p[1]
Enterococci	1 (2.44)	Enterococci	21 (10.40)	0.48
Fungi	0 (0.00)	Fungi	2 (1.0)	0.84
HACEK	3 (7.32)	HACEK	6 (3.0)	0.70
Staphylococcus aureus	14 (34.15)	*Staphylococcus aureus*	41 (20.3)	0.05
Streptococcus bovis	1 (2.44)	*Streptococcus bovis*	10 (5.0)	0.79
Other streptococci	2 (4.88)	Other streptococci	23 (11.4)	0.72
Viridans group	10 (24.39)	Viridans group	45 (22.3)	0.78
Other	7 (17.07)	Other	40 (19.8)	0.66
Negative culture findings	3 (7.32)	Negative culture findings	14 (6.9)	1.0
Total	41 (100)	Total	202 (100)	202 (100)

[1] before IPTW

Table S2. Patient characteristics before inverse probability of treatment weighting.

**Patient Characteristics**	**HA (*n*=41)**	**Control (*n*=200)**	**stddiff**	***p***
Age, years	59.0 ± 15.4	66.8 ± 13.6	−0.537	0.001
Female	5 (12.2%)	45 (22.5%)	0.275	0.145
BMI	25.9 ± 4.8	25.9 ± 5.4	−0.003	0.988
Ejection fraction, %	55.8 ± 12.0	56.2 ± 10.3	−0.033	0.841
Diabetes	9 (22.0%)	40 (20.0%)	−0.048	0.777
Current Smoker	10 (24.4%)	47 (23.5%)	−0.021	0.903
Platelet aggregation inhibitor	29 (70.7%)	110 (55.0%)	−0.330	0.067
Peripheral artery disease	5 (12.2%)	15 (7.5%)	−0.158	0.326
Preoperative stroke	15 (36.6%)	61 (30.5%)	−0.129	0.446
Renal disease	6 (14.6%)	31 (15.5%)	0.024	0.889
Dialysis	3 (7.3%)	9 (4.5%)	−0.120	0.454
COPD	2 (4.9%)	16 (8.0%)	0.127	0.493
Hypertension	16 (39.0%)	108 (54.0%)	0.304	0.083
Hypercholesteremia	10 (24.4%)	64 (32.0%)	0.170	0.338
NYHA III or IV	11 (26.8%)	94 (47.0%)	0.427	0.020
Preoperative AF	2 (4.9%)	26 (13.0%)	0.288	0.157
Prior MI	4 (9.8%)	10 (5.0%)	−0.183	0.244
Emergency	7 (17.1%)	24 (12.0%)	−0.144	0.379
EuroSCORE II, %	6.4 (4.3 to 9.3)	9.4 (7.9 to 11.1)	0.195	0.080
CRP, mg/L	0.6 (0.1 to 3.2)	0.2 (0.1 to 0.5)	0.009	0.175
Fibrinogen, g/L	3.0 (2.7 to 3.3)	2.6 (2.3 to 3.0)	0.591	0.348
Hemoglobin, g/L	66.2 (38.3 to 114.5)	62.3 (47.4 to 81.7)	0.164	0.978
WBC, counts/nl	11.0 (6.8 to 17.7)	10.2 (8.1 to 12.9)	0.212	0.658
Platelets, counts/nl	116 (64.3 to 210)	113 (84.2 to 152)	0.136	0.515

HA: hemoadsorption group; stddiff: standardized difference; BMI: body mass index; AF: Atrial fibrillation; COPD chronic obstructive pulmonary disease; NYHA New York Heart Association Functional Classification; MI myocardial infarction; EuroSCORE II European System for Cardiac Operative Risk Evaluation; CRP: C-reactive protein; WBC: white blood cell count.

Table S3. Perioperative details before inverse probability of treatment weighting.

**Perioperative Details**	**HA (*n*=41)**	**Control (*n*=200)**	**stddiff**	***p***
Perfusion time, min	135.6 (115.1 to 159.9)	138.4 (130.4 to 146.9)	0.604	0.692
Aortic clamping time, min	109.3 ± 59.5	107.1 ± 47.9	0.040	0.801
IV inotropes before surgery	7 (17.5%)	40 (20.0%)	0.064	0.716
Aortic valve	30 (73.2%)	145 (72.5%)	−0.015	0.930
Mitral valve	17 (41.5%)	93 (46.5%)	0.102	0.556
Tricuspid valve	2 (4.9%)	11 (5.5%)	0.028	0.872
Severe insufficiency	10 (24.4%)	59 (29.5%)	0.115	0.510
Procedure Groups				0.568
• CABG & Valve(s)	2 (4.9%)	20 (10.0%)	−0.196	
• CABG & Valve(s) & Other	2 (4.9%)	16 (8.0%)	−0.127	
• Valve(s) & Other	19 (46.3%)	76 (38.0%)	0.170	
• Valve(s) only	18 (43.9%)	88 (44.0%)	−0.002	
Assist Device				0.008
• IABP	2 (4.9%)	10 (5.0%)	−0.006	
• ECMO	3 (7.3%)	1 (0.5%)	0.357	

HA: hemoadsorption group; stddiff standard difference IV: Intravenous; CABG: Coronary artery bypass grafting; IABP: intraaortic balloon pump; ECMO: extracorporeal membrane oxygenation.

Table S4. Intensive care unit (ICU) data before inverse probability of treatment weighting.

**Intensive Care Unit Data**	**HA (*n*=41)**	**Control (*n*=200)**	**stddiff**	***p***

Administration of				
• Epinephrine	21 (51.2%)	89 (44.5%)	−0.135	0.432
• Dobutamine	1 (2.5%)	6 (3.1%)	0.039	0.832
• Milrinone	11 (27.5%)	36 (18.2%)	−0.222	0.195
• Nitroglycerine	3 (7.5%)	29 (14.5%)	0.224	0.252
• Norepinephrine	33 (80.5%)	105 (52.5%)	−0.621	0.002
• RBC	23 (56.1%)	62 (31.0%)	−0.523	0.003
• Tranexamic acid	1 (2.5%)	9 (4.4%)	0.103	0.593
• Haemate	4 (10.0%)	8 (3.8%)	−0.248	0.121
• FFP	23 (56.1%)	51 (25.5%)	−0.655	<0.001
• Fibrinogen	8 (20.0%)	31 (15.7%)	−0.112	0.517
• PCC	5 (12.5%)	26 (13.2%)	0.021	0.906
• Platelets	11 (26.8%)	20 (10.0%)	−0.445	0.005
Intubation >72h	1 (2.4%)	21 (10.5%)	0.332	0.137
Drainage >800ml within 12h	12 (30.0%)	50 (25.0%)	−0.112	0.521
Length of ICU stay, days	4.6 (3.3 to 6.5)	3.3 (2.8 to 3.9)	0.439	0.284
Postoperative renal failure	7 (17.1%)	42 (21.0%)	0.100	0.570
RRT	4 (9.8%)	14 (7.0%)	−0.100	0.543
Reoperation for bleeding	8 (19.5%)	16 (8.0%)	−0.339	0.030
Reoperation later than 24h	2 (4.9%)	10 (5.0%)	0.006	0.974

HA: hemoadsorption group; stddiff standard difference; RBC: red blood cell concentrates; FFP: fresh frozen plasma; PCC: prothrombin complex concentrate; ICU intensive care unit; RRT: renal replacement therapy.

Table S5. Postoperative details before inverse probability of treatment weighting.

Postoperative Details	HA (*n*=41)	Control (*n*=200)	stddiff	*p*
AF at discharge	15 (36.6%)	52 (26.0%)	−0.230	0.171
Delirium	15 (36.6%)	55 (27.5%)	−0.196	0.245
In-hospital mortality	5 (12.2%)	22 (11.0%)	−0.037	0.825
Length of stay	14.2 (10.7 to 18.8)	8.8 (6.9 to 11.3)	0.399	0.027
MACCE	7 (17.1%)	29 (14.5%)	−0.071	0.674
Neurological complication	16 (40.0%)	59 (29.7%)	−0.216	0.206
Permanent pacemaker	4 (9.8%)	41 (20.5%)	0.303	0.117
Pulmonary infection	5 (12.2%)	17 (8.5%)	−0.122	0.457
Postoperative sepsis	7 (17.1%)	13 (6.5%)	−0.332	0.031
Postoperative stroke	2 (4.9%)	9 (4.5%)	−0.018	0.916
Renal replacement therapy	4 (9.8%)	14 (7.0%)	−0.100	0.543

HA: hemoadsorption group; stddiff standard difference; AF Atrial fibrillation; MACCE Major adverse cerebrovascular and cardiac events.

Table S6. Association of hemoadsorption (HA) therapy and biomarker development before inverse probability of treatment weighting. We found that HA only showed an association with a decrease of white blood cell counts on the first postoperative day in patients who had undergone HA treatment, which evened out until day five.

Parameter	HA Coefficient (95% CI)	*p*	Time, days Coefficient (95% CI)	*p*	Interaction Coefficient (95% CI)	*p*
**Day 1 to 2**						
CRP	−3.34 (−54.4 to 47.7)	0.898	89.1 (80.2 to 97.9)	<0.001	2.17 (−30.6 to 35.0)	0.897
Fibrinogen	0.17 (−0.21 to 0.55)	0.389	0.70 (0.60 to 0.80)	<0.001	−0.04 (−0.24 to 0.16)	0.691
Hemoglobin	−1.71 (−7.73 to 4.31)	0.578	−3.99 (−5.47 to −2.51)	<0.001	−0.12 (−3.32 to 3.08)	0.941
WBC	−4.03 (−6.93 to −1.14)	0.006	−0.69 (−1.19 to −0.19)	0.007	2.28 (0.59 to 3.98)	0.008
Platelets	−9.66 (−49.8 to 30.5)	0.637	−4.03 (−9.07 to 1.00)	0.117	5.51 (−13.5 to 24.5)	0.571
**Day 1 to 3**						
CRP	−4.82 (−47.7 to 38.1)	0.826	41.8 (35.8 to 47.9)	<0.001	2.88 (−18.5 to 24.2)	0.791
Fibrinogen	−0.03 (−0.43 to 0.37)	0.890	0.49 (0.41 to 0.56)	<0.001	0.08 (−0.09 to 0.24)	0.356
Hemoglobin	−4.33 (−9.62 to 0.95)	0.108	−2.79 (−3.68 to −1.91)	<0.001	1.36 (−0.87 to 3.59)	0.231
WBC	−3.94 (−6.33 to −1.55)	0.001	−0.63 (−1.05 to −0.21)	0.003	1.78 (0.62 to 2.93)	0.003

Platelets	−10.0 (−44.8 to 24.7)	0.572	3.50 (−0.77 to 7.77)	0.108	4.28 (−8.17 to 16.7)	0.500
			**Day 1 to 5**			
CRP	−3.96 (−34.9 to 27.0)	0.802	1.61 (−1.60 to 4.81)	0.326	1.96 (−7.97 to 11.9)	0.699
Fibrinogen	−0.08 (−0.50 to 0.34)	0.700	0.30 (0.25 to 0.35)	<0.001	0.08 (−0.04 to 0.20)	0.195
Hemoglobin	−1.43 (−6.46 to 3.59)	0.576	−0.29 (−0.81 to 0.23)	0.268	−0.28 (−1.90 to 1.34)	0.732
WBC	−2.20 (−4.04 to −0.35)	0.020	−0.46 (−0.68 to −0.23)	<0.001	0.63 (0.04 to 1.22)	0.035
Platelets	6.74 (−23.9 to 37.4)	0.666	17.0 (13.4 to 20.5)	<0.001	−1.90 (−11.7 to 7.94)	0.706

CI: confidence interval; CRP: C-reactive protein; WBC: white blood cell count.
